# Outcomes and Challenges of Allograft Reconstruction for Distal Tibial Tumors: A Retrospective Case Series

**DOI:** 10.7759/cureus.109246

**Published:** 2026-05-19

**Authors:** Wan Yuhana W Md Yusof, Sanjeevan Radhakrishnan, Ang JS, Azuhairy Azid

**Affiliations:** 1 Orthopaedic Surgery, Penang General Hospital, George Town, MYS; 2 Orthopaedic Surgery, Kementerian Kesihatan Malaysia (KKM), Putrajaya, MYS; 3 Orthopaedic Surgery, Hospital Cancelor Tuanku Muhriz, Kuala Lumpur, MYS; 4 Orthopaedics and Traumatology, Penang General Hospital, George Town, MYS

**Keywords:** allograft reconstruction, ankle arthrodesis, functional outcome, limb salvage surgery, osteoarticular reconstruction, tibial tumor

## Abstract

Background

Reconstruction of distal tibial tumours remains technically challenging due to limited soft tissue coverage, complex ankle biomechanics, and the need to achieve oncological clearance while preserving limb function. Although allograft reconstruction represents a biological limb salvage option, evidence regarding its outcomes in distal tibial reconstruction remains limited. This study aimed to evaluate graft union, complications, graft survival, oncological outcomes, and functional outcomes following tibial allograft reconstruction.

Methods

A retrospective observational study was performed at a tertiary orthopaedic oncology centre, reviewing patients who underwent tibial allograft reconstruction between 2016 and 2024. Six patients with distal or diaphyseal tibial tumours were included. Outcomes assessed included graft union, complications, graft survival, oncological outcomes, and functional outcomes measured using the Musculoskeletal Tumor Society (MSTS) score.

Results

Mean time to union was 19 months (range: 9-24 months). Five patients developed complications, including deep infection in three, non-union in one, and allograft fracture in one. Four patients required revision surgery. At final follow-up, three of six grafts remained intact. Mean MSTS score was 63.9% (range: 33.3%-96.7%). No local recurrence was observed.

Conclusion

Allograft reconstruction for distal tibial tumours may be considered a limb salvage option in carefully selected patients; however, it is associated with substantial complication and revision rates. Careful patient selection and continued refinement of reconstructive strategies are essential to improve outcomes while preserving limb function.

## Introduction

Reconstruction of the distal tibia presents a unique challenge in orthopaedic oncology. Due to its subcutaneous location, limited soft tissue coverage, and proximity to the neurovascular bundle, achieving adequate oncological margins while preserving limb function can be difficult [1]. In addition, restoration of ankle biomechanics and stability following reconstruction remains technically demanding [2]. Historically, amputation was the main surgical treatment to ensure oncological clearance and minimize the risk of recurrence [3,4]. However, amputation is associated with substantial physical limitation and psychological burden [2,5].

With advances in chemotherapy, reconstructive techniques, and surgical planning, limb salvage surgery has become a viable option for patients with primary malignant or aggressive benign bone tumours of the distal tibia. Non-biological reconstruction represents one strategy, including options ranging from three-dimensional printed implants to custom-made prostheses and endoprostheses [1,6]. These methods offer the advantages of early functional recovery, improved ambulation, and preservation of ankle range of motion (ROM). However, long-term functional deterioration may occur due to implant-related complications, such as aseptic loosening, fibular impingement, talar collapse, and deep infection. Furthermore, the high cost and limited availability of custom-made implants and endoprostheses may restrict their use in certain healthcare settings.

Biological reconstruction is another feasible option and includes recycled autograft, allograft reconstruction with or without vascularised fibular graft (VFG) augmentation, and distraction osteogenesis to restore osseous defects and limb length [4,6-8]. Although biological reconstruction generally requires a longer period for functional recovery and delayed weight bearing, it may provide superior long-term durability and function compared with prosthetic reconstruction. However, these techniques are also associated with complications, including deep infection, graft resorption, fracture, and donor-site morbidity in cases involving VFG harvest.

This study describes the real-world challenges and complications associated with allograft reconstruction in a heterogeneous cohort of tibial tumour cases at our centre. Specifically, we assessed graft union, complications, and functional outcomes, with the aim of identifying factors influencing reconstructive success.

This case series was previously presented as an oral presentation at the Asia Pacific Musculoskeletal Tumor Society (APMSTS) meeting in October 2025.

## Materials and methods

Study design and patient selection

A retrospective observational study was performed at a tertiary orthopaedic oncology referral centre. This study was conducted in accordance with institutional guidelines for retrospective clinical research using anonymized data.

All consecutive patients who underwent tibial allograft reconstruction between 2016 and 2024 were included. Pathological diagnosis was confirmed by core needle biopsy. Allograft reconstruction was selected in cases with extensive bone involvement where recycled autograft was not feasible, and prosthetic reconstruction options were not available in our setting.

Treatment and postoperative care

All allografts used were fresh-frozen grafts obtained from the University Malaya Medical Centre (UMMC) bone bank, which follows standardized preparation, packaging, and delivery protocols. The allografts were prepared by soaking in diluted iodine solution, followed by vancomycin-infused sterile water, and subsequently thawed in warm normal saline immediately prior to implantation.

Perioperative intravenous antibiotics were administered at induction and continued postoperatively according to institutional protocol. An anterolateral incision was used in all cases. Following tumour resection, the allograft was fashioned according to the osteotomy length. In five patients, ankle arthrodesis was performed by preparing the distal surface of the allograft and the superior surface of the talus through cartilage removal and subchondral bone perforation. The ankle was subsequently aligned in a neutral position (90°) with slight external rotation.

Reconstruction was stabilised using tibial locking compression plates (LCP) in five patients, while one patient underwent hindfoot nail fixation due to a low osteotomy level (<3 cm from the joint).

Postoperatively, the ankle joint was immobilised in a splint for six weeks to protect fixation and facilitate arthrodesis. Physiotherapy for muscle strengthening and preservation of the range of motion of adjacent joints was initiated immediately. Non-weight-bearing mobilization was maintained until radiological evidence of callus formation at the proximal host-allograft junction and tibiotalar fusion was observed.

Rehabilitation, follow-up routine, and outcome measurement

Partial weight bearing was permitted once radiological evidence of callus formation was observed, and full weight bearing was allowed after complete union was achieved.

Union was assessed on serial anteroposterior and lateral radiographs. Radiological union was defined as diminished visibility of the osteotomy line or restoration of continuity in at least three out of four cortices [6,9].

Patients were reviewed monthly for the first three months postoperatively, followed by six-weekly and subsequently three-monthly reviews, with further intervals adjusted according to clinical progress.

Complications, including local recurrence, wound complications, infection, allograft fracture, and implant failure, were documented and managed accordingly. Deep infection in this series was defined according to the Centers for Disease Control and Prevention (CDC) National Healthcare Safety Network (NHSN) criteria for deep incisional or organ/space surgical site infection [10]. Serial metastatic surveillance was performed in patients with primary bone sarcoma. Clinical or radiographic findings suspicious for local recurrence were further investigated with MRI.

Functional outcome was assessed using the Musculoskeletal Tumor Society (MSTS) score [11]. Complications and graft survival were recorded and analysed descriptively without formal statistical comparison.

## Results

A total of six patients underwent tibia allograft reconstruction, comprising two males and four females. Histopathological diagnoses include three cases of primary malignant bone tumor: one each of osteosarcoma, Ewing sarcoma, and chondrosarcoma. One patient had leiomyosarcoma with close tibial involvement, and two patients were diagnosed with giant cell tumor (GCT) of the bone (Table 1). None of the patients presented with a pathological fracture. Patient demographics and surgical characteristics are summarized in Table 1.

**Table 1:** Patient demographics and surgical characteristics

No	Age	Sex	Diagnosis	Site	Union (mo)	Complication (mo)
1	33	F	Chondrosarcoma	Distal tibia	9	-
2	45	F	GCT	Distal tibia	24	74
3	41	M	GCT	Distal tibia	-	13
4	21	F	Ewing sarcoma	Intercalary	-	10
5	54	F	Osteosarcoma	Distal tibia	24	14
6	27	M	Leiomyosarcoma with tibia involvement	Distal tibia	-	6

The mean age at presentation was 30.3 years (range: 21-54 years). Five tumors were located in the distal tibia, while one was located in the diaphysis. Two patients received neoadjuvant chemotherapy (osteosarcoma and Ewing sarcoma) according to institutional oncology protocols. The patient with leiomyosarcoma received postoperative chemotherapy following the detection of pulmonary metastases on pulmonary CT surveillance. None of the patients received preoperative or postoperative radiotherapy. Neither of the two patients with GCT received preoperative denosumab.

The follow-up duration ranged from 17 to 108 months (Table 2). The shortest follow-up occurred in the patient who succumbed to disease progression at 17 months postoperatively. No other patients were lost to follow-up.

**Table 2:** Complications, management, and clinical outcomes AKA = above-knee amputation; AWD = alive with disease; DOD = died of disease; LLRS = limb length reconstruction system; NED = no evidence of disease

No	Complication	Treatment	Outcome	Removal	MSTS (%)	Oncologic	Follow-up
1	None	None	Union	No	96.7	NED	9y
2	Infection	Debridement + curettage	Union	No	63.3	NED	9y
3	Infection	Debridement, removal allograft, LLRS+ bone transport, ankle fusion	Healed	Yes	73.3	NED	8.5y
4	Fracture	None	Non-union	No	36.7	DOD	17m
5	Non-union	Plating + bone graft	Union	No	80	NED	3y
6	Infection	Debridement, antibiotics, AKA	Healed	Yes	33.3	AWD	18m

Five of six patients developed complications. Complications, management, and clinical outcomes are summarized in Table 2. Final graft union was achieved in three of six patients, with a mean time to union of 19 months (range: 9-24 months) (Table 1), including one patient who required revision fixation and bone grafting for initial non-union. The only patient without complications achieved union at nine months and recorded the highest MSTS score of 96.7%.

Deep infection occurred in three patients (Table 2). One patient developed an infection during the eighth postoperative year and underwent debridement with bone curettage; her allograft had previously united at 24 months. Another patient underwent staged debridement, allograft removal, and insertion of an antibiotic cement spacer, followed by distraction osteogenesis to reconstruct the defect with docking at the tibiotalar joint. Seven years after union at the docking site, this patient required further debridement for infection at the ankle arthrodesis site. The third patient with deep infection underwent transfemoral amputation 10 months after the index surgery.

Non-union at the proximal host-allograft junction occurred in one patient (Table 2), who had undergone hindfoot nail fixation. This was treated with additional plating and bone grafting, with union subsequently achieved at 24 months.

An allograft fracture occurred in one patient. No revision surgery was performed because the patient developed progressive pulmonary metastases and subsequently succumbed to the disease 17 months after surgery.

Four patients required revision surgery, although only one patient required more than one revision procedure. No local recurrence was observed in any patient.

The overall MSTS score at final follow-up was 63.9% (range: 33.3%-96.7%) (Table 2), representing final functional outcomes after all interventions. At final follow-up, three of six allografts remained in situ. Patients who achieved graft union and retained their allograft generally demonstrated higher functional scores, whereas those who developed complications, particularly infection or graft failure, had poorer outcomes. This highlights the impact of complications on functional outcome in this cohort.

## Discussion

Distal tibial bone tumors are rare, and the literature evaluating reconstructive strategies at this anatomical site remains limited. Our study adds to the existing evidence regarding allograft reconstruction in this uncommon clinical scenario. However, interpretation of the present findings is limited by the retrospective design, small cohort size, and lack of a comparison group. Given the rarity of these tumors, the heterogeneous nature of our cohort reflects real-world clinical practice. No subgroup comparison was attempted due to the limited number of patients. As alternative reconstructive options such as VFG or endoprosthetic reconstruction were not routinely performed at our centre, direct comparison was not possible. Furthermore, surgical method selection was influenced by tumor characteristics and surgeon preference, introducing potential selection bias.

Amputation has historically been, and remains, a viable option to ensure adequate negative margins during distal tibial tumor surgery. In addition to lower risks of local recurrence and wound complications, its functional outcome is only slightly inferior to reconstruction (MSTS of 70% vs 77%) [8]. However, amputation may impose substantial physical, psychosocial, and emotional burden on patients [12]. Several reconstructive methods have been described in the literature. Recycled autograft offers the advantages of exact anatomical matching, low cost, no risk of disease transmission, and suitability in centres where allograft or prosthetic reconstruction is unavailable. Although some studies demonstrated no significant difference between recycled autograft and allograft reconstruction [13,14], a systematic review by Zhao et al. reported superior functional outcomes and fewer complications with recycled autografts [8].

Distraction osteogenesis is another biological reconstruction option. Patients have been reported to achieve early full weight-bearing and favorable functional outcomes at final follow-up. However, prolonged duration of Ilizarov fixator use, the requirement for secondary removal procedures, and complications such as pin-site infection, delayed union, malunion, and docking-site problems must be considered. In addition, the consolidation period is prolonged, resulting in an increased bone healing index, particularly in patients receiving chemotherapy during bone transport [8].

Prosthetic reconstruction offers immediate structural stability, earlier ambulation, preservation of ankle joint function, and the ability to reconstruct large defects. However, prosthesis-related complications include mechanical failure, aseptic loosening, and talar collapse. Although many studies have demonstrated superior short- and mid-term functional outcomes with prosthetic reconstruction, these outcomes tend to deteriorate over time, negatively affecting long-term implant survival [1,2,8,13]. More recently, three-dimensional (3D)-printed implants have shown promising early functional outcomes at three years, with fewer complications than modular prostheses [3]. These implants have been associated with lower risks of infection, wound complications, and mechanical failure. Although failures are most commonly observed within the first postoperative year, successful revision surgery or conservative management may allow long-term durability due to the titanium material properties and controlled implant porosity [3,15].

Allograft reconstruction restores the osseous defect following tumor resection and is particularly useful in paediatric patients, as it does not significantly affect growth when only a single physis is involved [6,16]. Consequently, limb length discrepancy is less problematic. Similar to other biological reconstructions, once incorporation and healing to the host bone occur, the construct can tolerate mechanical stress for many years. During the first one to two years, the allograft is mechanically weaker due to repeated resorption and revascularisation during creeping substitution, predisposing it to complications during this period [13]. This was reflected in the present series, where all but two patients developed complications within two years of surgery. Notably, the only patient without complications achieved early union and the highest functional score, whereas patients who developed infection or graft failure demonstrated poorer functional outcomes. This reinforces the importance of successful graft incorporation and complication avoidance in determining overall reconstructive success.

Reported complication rates for allograft reconstruction range from 28% to 39% [8,17], with some studies reporting rates as high as 92% [4,18]. Common complications include infection, allograft fracture, non-union, and wound complications [4,6,8,13]. In contrast, Xu et al. reported only minor complications, including subcutaneous fluid collection and screw breakage [7]. Our series demonstrated a higher infection rate (50%) than that reported in most previous studies, although the overall pattern of complications was otherwise similar. This may be attributable to chemotherapy-related immunosuppression [13], limited soft tissue coverage around the distal tibia, and the absence of adjunctive soft tissue procedures in our cohort. No local recurrence was observed in our cohort, suggesting satisfactory oncological control following limb salvage in this selected series.

In the present study, the high complication rate and modest graft survival suggest that allograft reconstruction in the distal tibia may function more as a temporising reconstructive strategy rather than a definitive long-term solution in certain settings. This is particularly relevant in centres where alternative options, such as endoprosthetic reconstruction or VFG, are not readily available. While limb salvage can be achieved, the substantial risk of infection, non-union, and revision surgery must be carefully considered during preoperative planning and patient counselling.

Beyond overall complication rates, several additional factors may influence outcomes following allograft reconstruction. There is conflicting evidence regarding whether graft location influences complication rates and outcomes. Some authors have suggested that anatomical location does not significantly affect outcome [4,6], except in hemicortical grafts, which appear to demonstrate superior functional outcomes with minimal complications [6,19]. They also reported that osteoarticular grafts, irrespective of anatomical site, perform worse than intercalary and hemicortical grafts due to higher rates of mechanical failure from subchondral collapse and joint instability. Therefore, preservation of part of the medial malleolus with intact deltoid ligament has been recommended to maintain ankle stability during osteoarticular reconstruction [18]. When symptomatic failure occurs, revision procedures, such as conversion to endoprosthesis or allograft-prosthetic composite, may be considered [19,20]. In selected cases, allograft reconstruction may be used to delay endoprosthetic reconstruction in younger patients.

Within this context, ankle arthrodesis is a commonly used reconstructive option. Previous studies have reported favorable oncological and reconstructive outcomes with ankle arthrodesis [21,22]. Sambri et al. reported lower complication rates in patients undergoing ankle arthrodesis (38%) compared with osteoarticular and intercalary reconstruction (91% and 65%, respectively), along with superior functional scores (median MSTS: 24 vs 22 for osteoarticular reconstruction) [4]. These findings are supported by other authors who similarly demonstrated improved functional outcomes following arthrodesis [12,18]. In their systematic review, Zhao et al. noted that most authors advocate arthrodesis for its excellent ankle stability, although equivalent functional outcomes may be achieved with ankle-preserving reconstruction when appropriately indicated [8]. In contrast, our findings demonstrated suboptimal functional outcomes with high complication rates, consistent with findings by Laitinen et al. [5]. Together with a 50% graft survival rate, this suggests that graft incorporation and complication burden are key determinants of long-term success. However, despite the use of ankle arthrodesis in most patients, outcomes remained limited in some cases due to complications such as infection and graft failure.

To address these challenges, augmentation with VFG has been proposed to improve union rates, reduce allograft fracture risk, and significantly lower infection rates compared with allograft reconstruction alone [13,16,23]. Ipsilateral pedicled VFG is beneficial in intercalary and selected intraphyseal resections [23]. However, for distal tibial reconstruction, free VFG may be more suitable. During the first two postoperative years, the allograft provides structural support, while the vascularised fibula is protected from stress fracture. Subsequently, fibular hypertrophy may compensate for progressive allograft weakening before eventual fusion occurs [22,23]. Nevertheless, VFG harvest carries disadvantages, including prolonged operative time, donor-site morbidity, contralateral leg morbidity, and the need for microsurgical expertise [8,21]. In our setting, the absence of VFG augmentation may have contributed to the observed complication rates, and its use could be considered as a potential strategy to improve graft incorporation and reduce complications.

## Conclusions

Reconstruction of distal tibial tumors remains challenging due to complex anatomy and the substantial risk of complications. Allograft reconstruction remains a limb salvage option in carefully selected patients; however, the high complication and revision rates observed in this study highlight the significant morbidity associated with this technique. It may, in some cases, serve as a temporary reconstructive strategy rather than a definitive long-term solution, particularly when alternative options are limited. Careful patient selection, meticulous surgical planning, and continued refinement of reconstructive strategies are essential to improve outcomes while preserving limb function. Future strategies should focus on reducing complications and improving graft incorporation to enhance long-term outcomes.
